# Effect of Modified Yukmijihwang-Tang on Sleep Quality in the Rat

**DOI:** 10.3390/clockssleep4020024

**Published:** 2022-05-27

**Authors:** SunYoung Lee, Hun-Soo Lee, Minsook Ye, Min-A Kim, Hwajung Kang, Sung Ja Rhie, Mi Young Lee, In Chul Jung, In-Cheol Kang, Insop Shim

**Affiliations:** 1 Department of Physiology, College of Medicine, Kyung Hee University, Seoul 02447, Korea; tramp68@nate.com (S.L.); jh486ms22@naver.com (M.Y.); 2 Department of Neuropsychiatry, College of Korean Medicine, Daejeon University, Daejeon 34520, Korea; 791009@gmail.com; 3 BioChip Research Center, Department of Biological Science, College of Biological Science, Hoseo University, Asan 31499, Korea; mkkim@hoseo.kr (M.-A.K.); hjkang@hoseo.kr (H.K.); 4 Department of Beauty Design, Halla University, Wonju 26404, Korea; sjlee@halla.ac.kr; 5 Korea Institute of Oriental Medicine, 1672 Yuseong-daero, Yuseong-gu, Daejeon 34054, Korea; mylee@kiom.re.kr

**Keywords:** Yukmijihwang-tang (Ym), electroencephalography (EEG), sleep, 5-HT2c receptor binding assay, ventrolateral preoptic nucleus (VLPO)

## Abstract

Many plants have been used in Korean medicine for treating insomnia. However, scientific evidence for their sedative activity has not been fully investigated. Thus, this study was carried out to investigate the sedative effects of the extracts of medicinal plants, including Yukmijihwang-tang and its various modified forms through the 5-HT2c receptor binding assay, and to further confirm its sleep-promoting effects and the underlying neural mechanism in rats utilizing electroencephalography (EEG) analysis. Enzyme-linked immunosorbent assay (ELISA) was used to measure serotonin (5-HT) in the brain. The water extracts of modified Yukmijihwang-tang (YmP) displayed binding affinity to the 5-HT2C receptor (IC_50_ value of 199.9 µg/mL). YmP (50 mg/kg) administration decreased wake time and increased REM and NREM sleep based on EEG data in rats. Additionally, treatment with YmP significantly increased the 5-HT level in the hypothalamus. In conclusion, the sedative effect of YmP can be attributed to the activation of the central serotonergic systems, as evidenced by the high affinity of binding of the 5-HT2C receptor and increased 5-HT levels in the brain of the rat. This study suggests that YmP can be a new material as a sleep inducer in natural products.

## 1. Introduction

Sleep is an indispensable component of health-related quality of life. Sleeplessness provokes dysregulation of homeostasis and increases vulnerability to inflammation-related diseases and chronic diseases [1,2]. Approximately 15% of the adult population suffers from chronic insomnia, and these estimates are even higher among adults with concomitant medical or psychiatric illnesses [3]. The classes of drugs commonly used for the treatment of insomnia include hypnotic medications, such as benzodiazepines, but they have been reported to be associated with adverse outcomes [4,5,6,7]. Therefore, interest in herbal drugs that improve sleep quality and avoid side effects is increasing as an alternative to prescription drugs. 

Korean traditional medicine has a long history of medical practices based on the cumulative knowledge of natural products as a source of therapeutic herbal drugs [8,9]. Several medicinal plants are used for improving sleep disturbance in Korea; however, there are only a few reports on their sedative effects. Yukmijihwang-tang (Ym) has the effect of restoring the blood circulation of the kidney by stabilizing the excited heart and promoting diuretics. For these reasons, Ym is selected as a drug to verify its sedative effects. This compound is composed of six herbs, *Rehmannia glutinosa*, *Cornus officinalis*, *Dioscorea batatas*, *Paeonia suffruticosa*, *Poria cocos*, and *Alisma orientale*. 

The molecular target for sedative activity has been mainly focused on regulating the serotonergic (5-HT) system, which is involved in various mental functions related to relaxation, stress, and sleep regulation [10,11,12]. The 5-HT2 (5-HT2A, 2B, 2C) receptor, generally known to be coupled with G-protein, has been reported to play a major role in various central nervous system functions such as mood, sleep, and satiety [13,14]. Particularly, the 5-HT2C receptor is reported to be the potential target for sedative or anxiolytic drugs [15,16,17,18,19]. 

The ventrolateral preoptic nucleus (VLPO) in the hypothalamus is the major area of the brain that acts as a sleep-promoting center and a molecular target of hypnotic medication [20]. Sleep-active neurons have been identified by immunohistochemical detection during sleep. Therefore, it is possible that the activation of the VLPO region indicates the initiation of the sleep state. It is known that neurons in the VLPO contain 5-HT and its receptors, which is one of the most important neurotransmitters due to its role in regulating sleep–wake homeostasis [21,22]. The sleep–wake state is assessed by evaluating shifts in electroencephalogram (EEG) frequencies. A continuous transition from the wake period to non-rapid eye movement (NREM) followed by rapid eye movement (REM) occurs [23]. Wakefulness is identified by the predominance of high frequency and low amplitude EEG. NREM is scored based on the presence of spindles interspersed with slow waves in the EEG. EEG power during REM is significantly reduced in lower frequency δ-wave and increased in the range of θ-wave activity. 

The objective of this study is to identify one herbal sedative candidate among modified forms of Yukmijihwang-tang through a 5-HT2c receptor binding assay and to confirm its sleep-promoting effects using animal sleep model experiments.

## 2. Results

### 2.1. 5-HT2c Receptor Binding Assay

**Figure 1:**
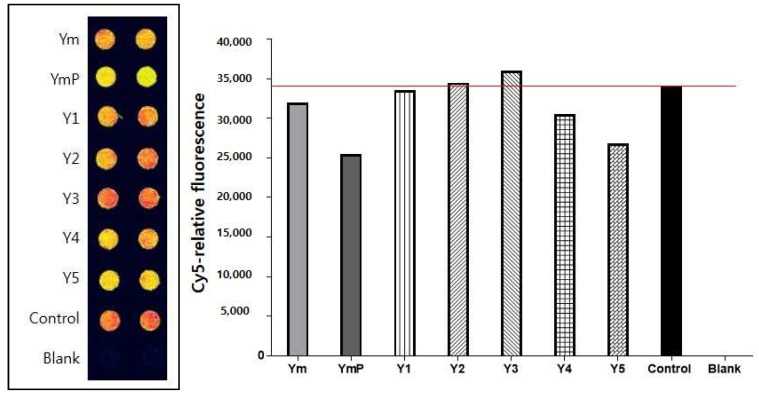
Screening of 5-HT2c receptor antagonists from modified Yukmijihwang-tang using protein chip-based 5-HT2cR assay. For screening effective antagonists to the 5-HT receptor, candidate drugs, including YmP, were reacted to a Protein Chip bound to 5-HT2c receptors, and then Cy5-tryptamine was added. In the case of YmP, the fluorescence signal was decreased, and YmP was an effective antagonist. Red color represents fluorescence intensity to Cy5-labeled tryptamine alone bound to 5HT2c receptors, and yellow color represents competitively drug-induced inhibitory binding activity. Concentration of 5-HT2cR is 50 μg/mL and concentration of Cy5-labeled Tryptamine is 500 μM. Yukmijihwang-tang (Ym); modified Yukmijihwang-tang (YmP), various modified forms of Yukmijihwang-tang (Y1-5).

**Figure 2:**
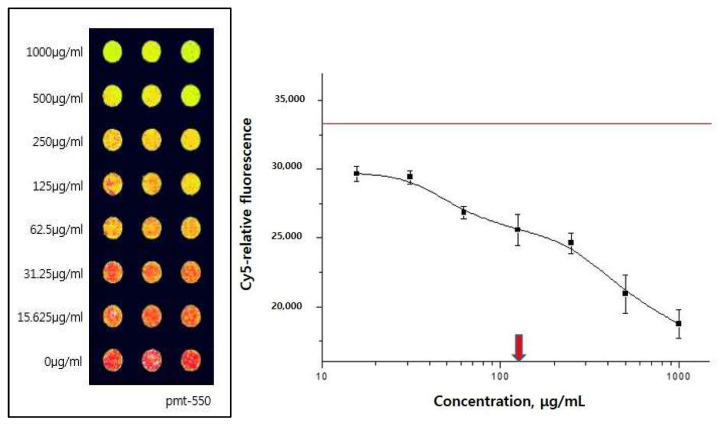
Dose-response curve and half-maximal inhibitory concentration (IC50) of YmP in the 5-HT2C receptor binding assay. When YmP was added to 5-HT2c receptors dose-dependently, the IC50 value of YmP was 199.9 µg/mL. Concentration of 5-HT2cR is 50 μg/mL and concentration of Cy5-labeled Tryptamine is 500 μM. Modified Yukmijihwang-tang (YmP); photomultiplier tubes (pmt).

The binding activities of modified Yukmijihwang-tang, YmP, to the 5-HT2C receptor were shown as 5-HT2C agonist binding. Results of screening of YmP appeared to be more effective for suppressing the interaction than Yukmijihwang-tang, Ym, and others (Figure 1). The YmP showed effective binding activity. The inhibitory concentration (IC50) value of YmP was 199.9 µg/mL (Figure 2).

### 2.2. Effect of YmP on EEG

**Figure 3:**
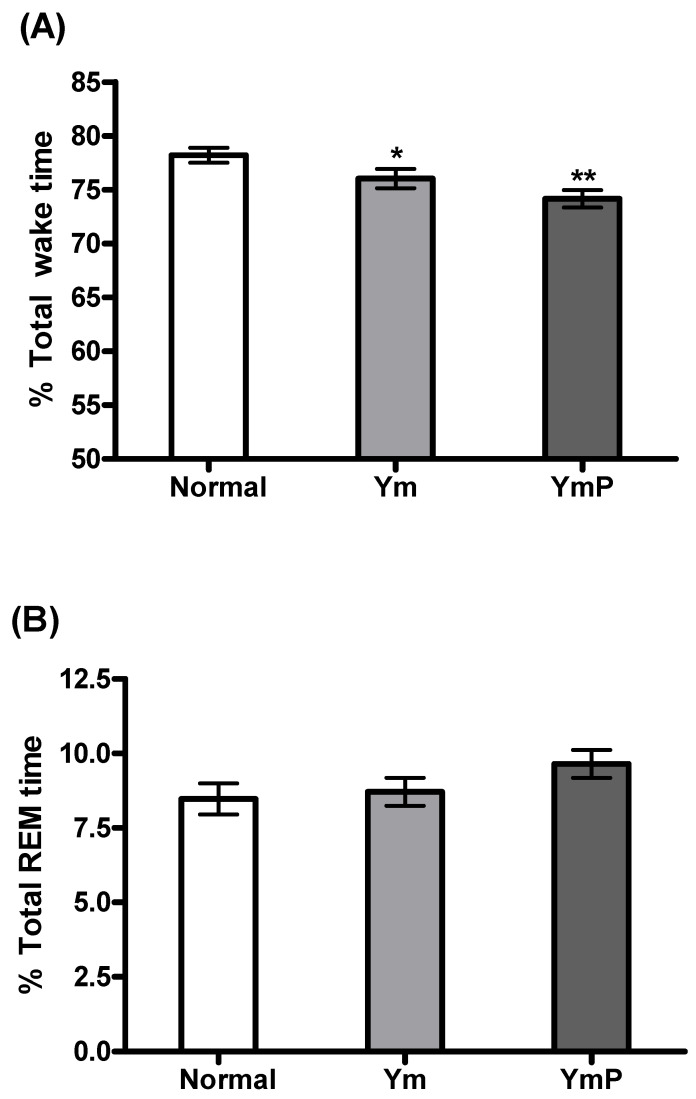

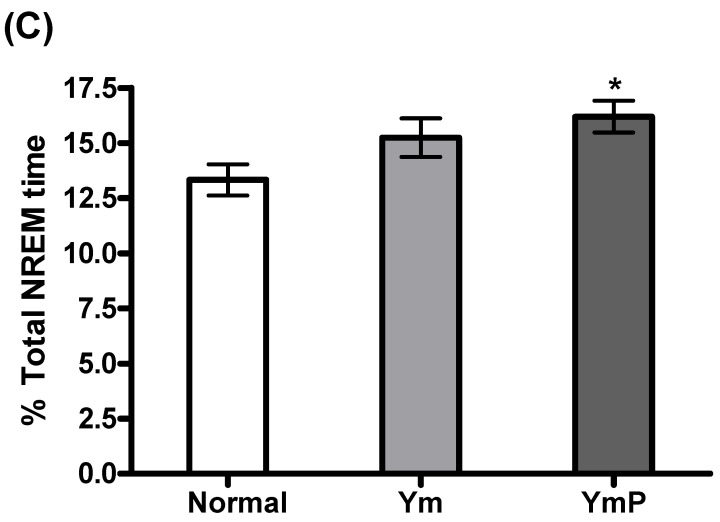
The effect of YmP on sleep architecture in the rat. Changes in the percentage of wake (**A**), REM sleep (**B**), and NREM sleep (**C**) during the dark phase are shown in the Ym and YmP-treated groups. The data represent the mean ± SEM of percent time spent in the sleep–wake state. * *p* < 0.05 vs. Normal (saline-treated group), ** *p* < 0.001 vs. Normal (saline-treated group); one-way ANOVA followed by Tukey HSD. Yukmijihwang-tang (Ym); modified Yukmijihwang-tang (YmP); rapid eye movement (REM); non-rapid eye movement (NREM).

The effect of YmP on EEG sleep architecture and profile was assessed (Figure 3). YmP treatment began at 8 p.m., and the EEG signals were recorded for the next 12 h. Ym and YmP significantly decreased wake time [F(2,23) = 9.341, *p* < 0.001, Figure 3A]. Ym did not produce any significant change in REM and NREM sleep, but a slight increase was shown compared to the saline-treated group (Figure 3B,C). YmP significantly increased NREM sleep [F(2,23) = 5.358, *p* < 0.05, Figure 3C] and tendency of increase in REM sleep was observed (Figure 3B). 

### 2.3. Effect of YmP on 5-HT Level in the Hypothalamus and PFC

**Figure 4:**
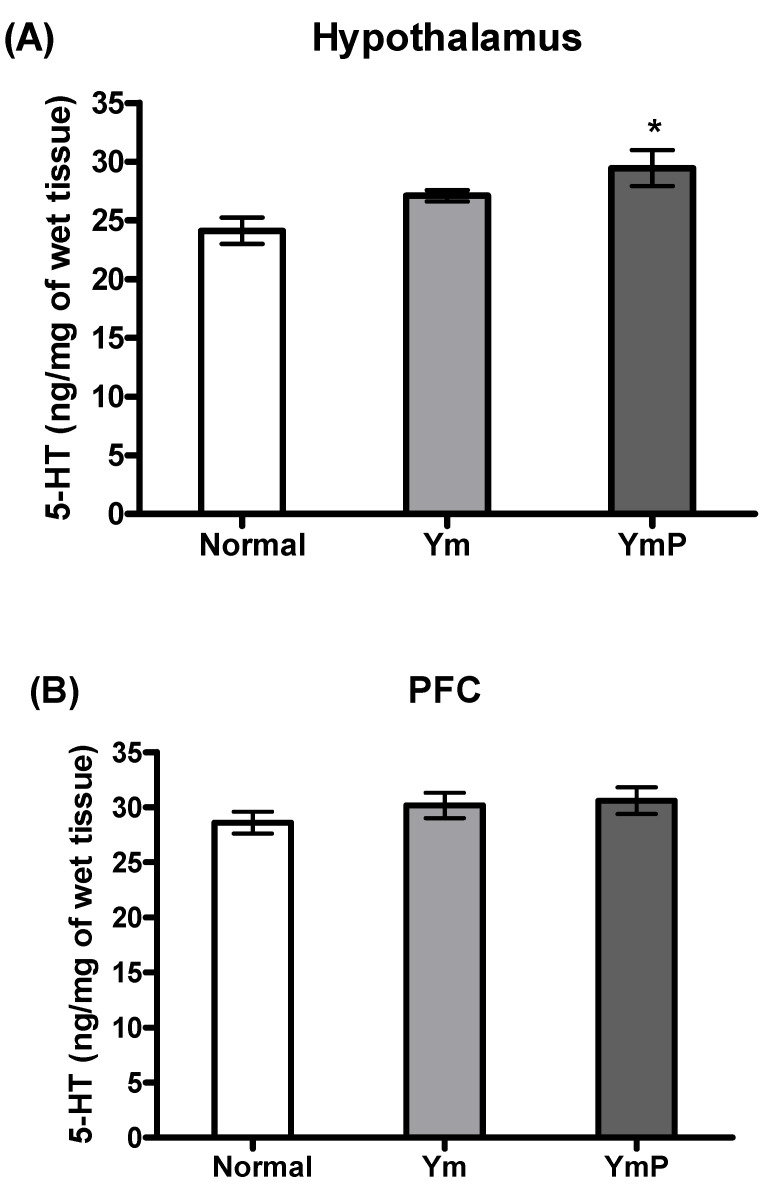
The effect of YmP on 5-HT concentration. Mean (± SEM) 5-HT level of the hypothalamus (**A**) and PFC (**B**) in the Ym and YmP treated groups. * *p* < 0.05 vs. Normal (saline-treated group); one-way ANOVA followed by Tukey HSD. Yukmijihwang-tang (Ym); modified Yukmijihwang-tang (YmP); prefrontal cortex (PFC).

The concentration of brain 5-HT was assessed in the Ym and YmP-treated groups (Figure 4). The YmP-treated group had significantly increased 5-HT levels in the hypothalamus region of the brain [F(2,17) = 5.601, *p* < 0.05, Figure 4A], but only a tendency of increment in the PFC region was observed (Figure 4A). The Ym-treated group tended to have increased 5-HT levels in the hypothalamus and PFC (Figure 4A,B).

## 3. Discussion

In the present study, Ym and its various modified forms used for the treatment of mental disorders were screened and selected to evaluate the sedative effects of their water extracts. First, this study investigated their binding affinity to the 5-HT2C receptor. Then, its sedative effects with high binding activity were evaluated using the analysis of sleep architecture and profile.

The receptor binding assay is an important tool in the search for drug candidates. Especially, the 5-HT2C receptor binding assay has been widely used to screen for sedative and anxiolytic activity. The screening test for Ym and various modified forms demonstrated that YmP showed the highest binding activity. YmP showed a moderate dose-dependent binding affinity to the 5-HT2C receptor (IC50 value of 199.9 µg/mL). This result indicated that YmP contained natural ligand binding affinity to the 5-HT2C receptor. 

Administration of YmP significantly reduced total wake time and increased NREM time. The 5-HT level in the hypothalamus was considerably increased in the YmP-treated group. The data showed the first experimental evidence that YmP has sedative efficacy, suggesting YmP is a novel natural medication to enhance sleep by modulating the serotonergic system and activating the sleep-promoting region in the brain. 5-HT depletion causes a significant increase of motor activity and modulation of the sleep–wake cycle [24]. Thus, it is suggested that the locomotor-reducing ability of YmP may contribute to sleep promotion through increasing 5-HT release. 

A newly created compound in which *Polygonum multiflorum* is added to the three herbs of Ym has been designated YmP. The three components of Ym, *Paeonia suffruticosa*, *Poria cocos*, and *Alisma orientale*, are known for reducing excessive heat and water in Korean medicine. *Polygonum multiflorum* has traditionally been used for palpitation, amnesia, and insomnia. Additionally, it has been shown that Drosophila is effective in an insomnia model [25]. 

Previous studies have reported that the administration of extracts from natural herbal remedies that exhibited a hypnotic effect altered total sleep time and NREM rather than REM sleep [26]. In accordance with these studies, the present research showed that Ym and YmP, respectively, reduced wake time, and YmP promoted significant NREM sleep. The central serotonergic systems have been implicated in promoting wakefulness [27], but their exact role in the sleep–wake system is still controversial. Extracellular 5-HT release during sleep deprivation was reduced [28] or increased [29]. Tryptophan, the precursor of 5-HT, was reported to improve sleep [22,30]. Another study suggested that 5-HT depletion-induced sleep disturbance occurs only under the condition of hypothermia [31].

In the present study, the 5-HT level in the hypothalamus and the PFC was slightly increased in both the Ym and YmP-treated groups. Hypothalamic VLPO neurons induce sleep [32]. There are two types of VLPO neurons that respond differently to 5-HT. 5-HT suppresses input to type 1 neurons, which have 5-HT 1, 2, 4, 7 receptors. Input to type 2 neurons containing 5-HT 2, 4, 7 receptors, however, is enhanced by 5-HT. Although two neurons are inversely influenced by 5-TH, they promote sleep by involving the initiation and maintenance of sleep, respectively [21]. It was also reported that the injection of 5-HT receptor 1A agonist increases wake time [33]. Accumulating evidence and the present results indicate that increasing 5-HT in VLPO may promote sleep through diverse 5-HT receptors located in type 1 and 2 neurons. 

The YmP-treated group showed significant changes in locomotor activity, wake–sleep state, and 5-HT concentration. It is assumed that an increased release of 5-HT in the hypothalamic VLPO neurons is sufficient to induce sedative behavioral changes. It has been shown that the hypothalamic VLPO neurons contain a variety of neurotransmitters, including 5-HT. The role of these 5-HT-containing neurons in regulating sleep behaviors is not clearly known, but it is possible that YmP may activate the central serotonergic neurons and increase release in the hypothalamic VLPO. Further studies examining the interaction of 5-HT receptors in the hypothalamic VLPO will interpret the exact neural mechanisms involved in sleep promotion. In addition, further studies are needed to investigate pharmacological and molecular mechanisms underlying the effect of YmP on 5-HT biosynthesis, its receptors, and interactions with other neurotransmitter systems. In the future, we also need to test the sleep-promoting effects of bioactive compounds among YmP, *Paeonia suffruticosa, Poria cocos, Alisma orientale, and Polygonum multiflorum* in animal sleep model experiments. 

Even though the present study demonstrated that oral administration of YmP decreased wake time and increased REM and NREM sleep and the central serotonergic activity, we did not evaluate or compare other routes of injections of YmP such as subcutaneous (SC) or intraperitoneal (IP) injections. In the present study, we did not assess whether YmP can cross the blood–brain barrier (BBB). Characteristics of YmP and its major compounds that cross the BBB are not known. These studies should be undertaken in the future.

Our data showed the first evidence that YmP has sedative efficacy, demonstrating YmP as a novel natural medication to enhance sleep by modulating the serotonergic system in the brain. It is suggested that YmP might be a useful natural alternative for hypnotic medicine. Our findings will benefit clinicians and researchers who are interested in complementary and alternative methods to improve sleep quality and provide valuable information for the development of clinical treatments or health food supplements to treat sleep disorders and maintain high-quality sleep in humans. 

## 4. Materials and Methods

### 4.1. Plant Material

The prescription and ratio of each component in Ym and YmP are shown in Table 1 and Table 2. The drug was purchased from Han Kook Shin Yak Pharmaceutical Co., Ltd. (Nonsan, Korea) (Table 1 and Table 2).

### 4.2. Preparation of Ym and YmP

YmP is composed of *Paeonia suffruticosa*, *Poria cocos*, *Alisma orientale*, and herbal medicine *Polygonum multiflorum* is added. Ym is Yukmijihwang-tang composed of *Paeonia suffruticosa*, *Poria cocos*, *Alisma orientale*, *Rehmannia glutinosa*, *Dioscorea batatas*, and *Cornus officinalis*. YmP and Ym were resuspended in water, heat-extracted for three hours, and filtered. The filtered fluid was then distilled at 60 °C using a rotary vacuum evaporator (Büchi 461, Eyela, Bohemia, NY, USA). Concentrated solutions were frozen at −70 °C and freeze-dried at −40 °C for 3 days. The dry weight of Ym and YmP extract was 11.05 g and 18.2 g, respectively, and their yields were 36.8% and 36.4%.

### 4.3. 5-HT2c Receptor Binding Assay

The 5-HT2c Receptor (Serotonin 5HT2c membrane preparation in HEK293 cells, Product #6110548400UA) was purchased from Perkin-Elmer (Walham, MA, USA). The Protein Chip was acquired from Proteogen, and Innopharmascreen Inc. Cy5-labeled Tryptamine was obtained from Peptron. Stock buffer for the 5-HT2c receptor was composed of 50mM Tris-HCL (pH 7.4), 0.5mM EDTA, 10 mM MgCl2, and 10% sucrose. The 5-HT2c receptor binding assay buffer was comprised of 50 mM Tris-HCl, 10 mM MgCl, 1mM EDTA, and 0.1% BSA pH 7.4.

The 5-HT2c receptor (50 µg/mL) in assay buffer (50 mM Tris-HCl, 10 mM MgCl, 1 mM EDTA, 0.1% BSA pH 7.4) was immobilized on the Protein Chip for 16 h at 4 °C as a substrate to capture protein. After washing in 0.05% PBST for 10 min twice and drying using N2 gas, the Protein Chip was blocked with 3% BSA for 1 h at room temperature. The 5-HT2c receptor microarray was then washed three times with PBST and dried. Cy5-labeled tryptamine (500 µM, using 30% glycerol in PBS as buffer) and YmP (using 30% glycerol in PBS as buffer) were applied to the Protein Chip and incubated for 1 h at 37 °C. The Protein Chip was then rinsed with PBST and DW and dried under a stream of N2 gas. YmP was dissolved in DW and diluted to the desired concentration using PBS. The YmP concentrations ranged from 1000 µM to 15.625 µg/mL. Tryptamine alone was used as a negative control. Various modified forms of Yukmijihwang-tang were used to assess the sedative effect through a 5-HT2c receptor binding assay.

### 4.4. Protein Chip Analysis

To detect fluorescence signals, the Protein Chip was scanned using a GenePix pro A4100 scanner and saved as a TIFF file. The scanning images were analyzed using GenePix pro 6.0 (Axon Instruments, Union City, CA, USA). Numerical data were processed using Excel (Microsoft, Redmond, WA, USA) and Origin 6.1 (Originlab, Northampton, MA, USA).

### 4.5. Animals

Adult male Sprague-Dawley rats (Samtako, Osan, Korea) weighing 250–300 g were used for the electroencephalography and 5-HT analysis in the brain. Rats were purchased from Samtako, Korea. The animals were housed in an air-conditioned room at a temperature of 20 °C–25 °C and a humidity of 45–65% under a 12:12 h light/dark cycle (lights on at 8 a.m.). Food and water were given ad libitum. For the care and use of laboratory animals, all the experimental procedures performed on the animals were conducted with the approval of the Ethics Committee of the Kyung Hee University (KHUASP(SE)-14-051) and in accordance with the US National Institutes of Health (Guide for the Care and Use of Laboratory Animals, 8th edition, revised 2011). In this study, the rats were randomly assigned to three groups: Normal group (*n* = 8); Ym group (*n* = 8); and YmP group (*n* = 8).

### 4.6. EEG Surgery

The animals were divided into three groups (normal; Ym, and YmP groups), with eight rats in each. Electroencephalogram (EEG) electrodes were implanted for polygraphic recording as described in the stereotaxic atlas of Paxinos and Watson [34]. Surgical anesthesia was achieved with pentobarbital (40 mg/kg, i.p.), after which rats were chronically implanted with a head mount. The body of the transmitter was implanted subcutaneously off the midline and posterior to the scapula attached to the skin and sutured three times for stabilization. The electrodes were anchored into the skull with screws and dental cement. All surgical procedures were performed stereotaxically under aseptic conditions. After surgery, each rat was placed in an individual transparent barrel for seven days for recovery.

### 4.7. Methodology of EEG Recording

After recovery, the rats were habituated to the recording conditions before the test. Water extracts of Ym and YmP were dissolved in 0.9% saline at a concentration of 50 mg/mL and then administered per os (p.o.) in a volume of 10 mL/1 kg of body weight for 5 days before EEG recording. Oral administration of saline and Ym, YmP were loaded 10 min before EEG recording. Recording began at 8:00 p.m., and 12 h of EEG and activity were recorded in all rats. Cortical EEG signals were amplified (×100), filtered (low-pass filter; 100 Hz EEG), digitized at a sampling rate of 200 Hz, and recorded with the PAL-8200 data acquisition system (Pinnacle Technology Inc., Lawrence, KS, USA) using a chart speed of 25 mm/s. 

### 4.8. EEG Data Analysis

The sleep–wake states were automatically classified into wakefulness (Wake), rapid eye movement (REM) sleep, and non-REM (NREM) sleep by SleepSign Ver. 3 software (Kissei Comtec, Nagano, Japan). Sleep latency was defined as the elapsed time between sample administration and the first consecutive NREM sleep episode lasting at least 2 min and uninterrupted by more than 64 s epochs, not scored as NREM sleep. 

### 4.9. 5-HT Measurement

After EEG recordings, the animals were deeply anesthetized with sodium pentobarbital (80 mg/kg, administered i.p.), and the brains were immediately removed and coronally sectioned by using the rodent brain matrix (ASI instruments Inc., Warren, MI, USA). The hypothalamus and prefrontal cortex (PFC) regions of the brain were punched out on a cold plate and stored at −70 °C until the assay. The obtained tissue was homogenized and incubated in an ice-cold protein extraction solution (iNtRON Biotechnology, Inc., Gyeonggi, Korea) for 30 min and centrifuged (10,000× *g* at 4 °C for 5 min). The supernatant was transferred to a fresh tube, and the 5-HT concentration in duplicate aliquots was assessed by an enzyme-linked immunosorbent assay (ELISA) kit according to the manufacturer’s instructions (Labor Diagnostika Nord, Inc., Minneapolis, MN, USA).

### 4.10. Statistical Analysis

All statistical analyses were conducted using SPSS (IBM^®^ SPSS^®^ Statistics Ver. 23). For multiple comparisons, the behavioral data were analyzed using one-way analysis of variance (ANOVA). Tukey’s post hoc test was used to identify significant differences among groups. The level of significance was set to *p* < 0.05.

## 5. Conclusions

The sedative effects of YmP against 5-HT2cR were investigated through a 5-HT2c receptor binding assay and detected by fluorescence signals using protein chip image analysis. A sleep test was then performed to examine the sleeping time of the rats. YmP blocked the 5-HT2c receptor-tryptamine interaction effectively and induced a significant decrease in total wake time and an increase in NREM and REM sleep and 5-HT levels in the brain. It is suggested that YmP might be a useful natural alternative for hypnotic medicine.

## Figures and Tables

**Table 1:** Prescription of Ym.

Galenic Name	Amount (g)
*Rehmannia glutinosa*	12.00
*Cornus officinalis*	8.00
*Dioscorea batatas*	8.00
*Paeonia suffruticosa*	6.00
*Poria cocos*	6.00
*Alisma orientale*	6.00
Total	46.00

**Table 2:** Prescription of YmP.

Galenic Name	Amount (g)
*Paeonia suffructicosa*	6.00
*Poria cocos*	6.00
*Alisma orentale*	6.00
*Polygonum multifluorum*	12.00
Total	30.00

## Data Availability

Not applicable.
